# Maternal predictors of neonatal outcomes after emergency cesarean section: a retrospective study in three rural district hospitals in Rwanda

**DOI:** 10.1186/s40748-017-0050-4

**Published:** 2017-06-13

**Authors:** Naome Nyirahabimana, Christine Minani Ufashingabire, Yihan Lin, Bethany Hedt-Gauthier, Robert Riviello, Jackline Odhiambo, Joel Mubiligi, Martin Macharia, Stephen Rulisa, Illuminee Uwicyeza, Patient Ngamije, Fulgence Nkikabahizi, Theoneste Nkurunziza

**Affiliations:** 1 Partners In Health, Inshuti Mu Buzima, Kigali, P.O. Box: 3432, Rwanda; 2 College of Medicine and Health Sciences, University of Rwanda, Kigali, Rwanda; 3 Department of Global Health and Social Medicine, Harvard Medical School, Boston, USA; 4 University of Colorado School of Medicine, Colorado, USA; 5 Brigham and Women’s Hospital, Boston, USA; 6 Partners In Health, Boston, USA; 7 Ministry of Health, Kigali, Rwanda

**Keywords:** Obstetric surgery, Emergency obstetrics, Birth outcomes, Africa

## Abstract

**Background:**

In sub-Saharan Africa, neonatal mortality post-cesarean delivery is higher than the global average. In this region, most emergency cesarean sections are performed at district hospitals. This study assesses maternal predictors for poor neonatal outcomes post-emergency cesarean delivery in three rural district hospitals in Rwanda.

**Methods:**

This retrospective study includes a random sample of 441 neonates from Butaro, Kirehe and Rwinkwavu District Hospitals, born between 01 January and 31 December 2015. We described the demographic and clinical characteristics of the mothers of these neonates using frequencies and proportions. We assessed the association between maternal characteristics with poor neonatal outcomes, defined as death within 24 h or APGAR < 7 at 5 min after birth, using Fisher’s exact test. Factors significant at α = 0.20 significance level were considered for the multivariate logistic regression model, built using a backwards stepwise process. We stopped when all the factors were significant at the α = 0.05 level.

**Results:**

For all 441 neonates included in this study, 40 (9.0%) had poor outcomes. In the final model, three factors were significantly associated with poor neonatal outcomes. Neonates born to mothers who had four or more prior pregnancies were more likely to have poor outcomes (OR = 3.01, 95%CI:1.23,7.35, *p* = 0.015). Neonates whose mothers came from health centers with ambulance travel times of more than 30 min to the district hospital had greater odds of having poor outcomes (for 30–60 min: OR = 3.80, 95%CI:1.07,13.40, *p* = 0.012; for 60+ minutes: OR = 5.82, 95%CI:1.47,23.05, *p* = 0.012). Neonates whose mothers presented with very severe indications for cesarean section had twice odds of having a poor outcome (95% CI: 1.11,4.52, *p* = 0.023).

**Conclusions:**

Longer travel time to the district hospital was a leading predictor of poor neonatal outcomes post cesarean delivery. Improving referral systems, ambulance availability, number of equipped hospitals per district, and road networks may lessen travel delays for women in labor. Boosting the diagnostic capacity of labor conditions at the health center level through facilities and staff training can improve early identification of very severe indications for cesarean delivery for early referral and intervention.

## Background

Globally, an estimated 22.9 million cesarean procedures are performed each year, primarily to save the life of either the mother or the infant [1]. However, at 1.77 deaths per 1,000 live births, global neonatal mortality post-cesarean section is three times higher than mortality following vaginal deliveries [2]. In sub-Saharan Africa, 8.8% of all deliveries are through cesarean section [3], falling within the 5–15% range recommended by the World Health Organization [4]. However, intrapartum neonatal mortality in sub-Saharan Africa accounts for 73% of global neonatal intrapartum deaths [5] and neonatal mortality post-cesarean delivery in sub-Saharan Africa is higher than the global average [3].

Poor neonatal outcomes post-cesarean delivery has been defined as mortality, low APGAR scores or admission to the neonatal intensive care unit [6, 7]. A variety of risk factors for poor neonatal outcomes have previously been identified. Shortage of staff, inadequately skilled staff and limited equipment impede timely availability of cesarean sections for women who need it [4]. A study in sub-Saharan Africa found that the majority of countries had limited capacity to provide cesarean sections, with only 20% of hospitals having full time physicians and 47% reporting a lack of anesthetists [8]. In addition, barriers such as the cost of cesarean delivery or delayed referral to the facility providing cesarean section also increase poor neonatal outcomes [4]. Fetal demographic characteristics such as fetal weight, fetal malformations and pre-operative fetal heart rate have also been linked to low APGAR scores [9, 10]. However, data on the effect of maternal factors on neonatal outcomes following cesarean section at district hospitals in Rwanda is limited.

In 2015 in Rwanda, the neonatal mortality rate was 20 per 1000 live births, approximately 30% of which occurred in the first 24 h of life [11]. About 13% of all deliveries are through cesarean section [11]. Of deliveries that need a cesarean section, 45% are performed at district hospitals [12] and at least 60% of all surgeries done at the district hospital are cesarean sections [13]. However, information on the neonatal outcomes immediately after cesarean section at these district hospitals or factors that are linked to poor neonatal outcomes is limited. In this study, we assessed the rates of poor neonatal outcomes and the maternal factors associated with poor neonatal outcomes after emergency cesarean delivery at three rural district hospitals in Rwanda. Identifying maternal predictors of poor neonatal outcomes could inform future interventions to improve neonatal survival post emergency cesarean section at the district hospitals.

## Methods

### Study setting

We conducted this study at Butaro, Kirehe and Rwinkwavu District Hospitals, three rural facilities that are operated by the Rwandan Ministry of Health with support from Partners In Health, a Boston-based non governmental organization. Rwinkwavu and Kirehe District Hospitals are located in the Eastern Province and Butaro District Hospital is located in the Northern Province of Rwanda. Together, the catchment area of these hospitals includes 46 health centers and serves more than 800,000 people [14]. These districts are in the rural settings where 49% of the population make 30 min travel to get to clean water source, and only 12% have access to electricity. The population in this setting is likely to work in small farming agricultural sector and 77% of poorer households work in agricultural sector compared to 21% of richer ones that work in the same sector [11].

According to Rwanda’s protocol on labor and delivery, pregnant women first present to the health center for delivery led by nurses. If the delivery is complicated to be managed at the health center, i.e. nurses trained in emergency obstetric care detect obstetrical emergencies such as mal-presentation, cord prolapse and others, then the mother is referred to the district hospital and transported using a Ministry of Health ambulance. District hospitals are equipped with obstetrical theatres, trained personnel and equipment to perform cesarean delivery, the recommended procedure for high-risk labor. Some health centers have their own ambulance (including 11 out of 46 health centers in the three study districts) and the remaining health centers call to the district hospital for assistance. After being admitted to the district hospital, a midwife or nurse will assess the pregnant woman and determine whether a cesarean section should be considered. A general practitioner makes the final decision for a cesarean section and leads the procedure.

In 2015, there were 7569 deliveries at the three district hospitals. Of all deliveries, 39% were through cesarean section and were considered high risk as they were referred from health centers due to complications or previous complications. More than 90% of deliveries are performed by skilled doctors, nurses and midwives [11]. These hospitals are staffed with 11–20 midwives, 73–90 nurses and 9–14 general practitioners. In the maternity department, two nurses or midwives staff the delivery room during the day or night. One general practitioner is assigned to delivery room during the day, but at night, one general practitioner services the whole of maternity department. Each of the three district hospitals is equipped with at least two operating tables, basic anesthesia equipment and supplies to deliver regional and general anesthesia, four obstetrics tables, thirty ward beds, six incubators and two lamps. A mother who has community-based health insurance is responsible for 10% of the total cost, with the remaining 90% paid by the government. Otherwise, the mother is responsible for all related delivery costs.

### Study design and population

This retrospective cross-sectional study included neonates born to mothers undergoing emergency cesarean section between 01 January and 31 December 2015 at Butaro, Kirehe and Rwinkwavu District Hospitals. Due to the large population of cesarean deliveries at the district hospitals and insufficient resources to collect data on all deliveries, we randomly sampled 200 cesarean deliveries per hospital.

The study’s inclusion criterion was emergency cesarean delivery without intrauterine death prior to the decision for cesarean section. Women who received elective cesarean section and those with non-emergent indications for cesarean section were excluded. For sample selection, because the majority of cesarean sections at district hospitals are due to the referral of complicated cases from health centers, we first assumed all cesarean section deliveries were emergency and used segmented sampling to sample 200 women in each hospital. Authors NN and YL led the sampling procedures. All cesarean section charts were grouped by month, a sampling interval determined and charts selected by skipping the interval. During sampling, women who had scheduled or requested for cesarean delivery (charts marked elective) and women with neonatal intrauterine death prior to the decision to conduct the cesarean section were removed and randomly replaced with other women who met inclusion criteria from same month of hospital admission. A total of 597 cesarean deliveries were sampled. During data cleaning, we excluded women with no indication that the cesarean delivery was truly emergent (this included women whose only indication was prior cesarean delivery and women who were post-term or with twins but no other emergent indication). The following diagnoses were considered emergent and included in the study: uterine rupture, fetal distress, cord prolapse, abruption placenta, preeclampsia prolonged rupture of membranes, cephalo-pelvic disproportion, prolonged labor, and mal-presentation.

### Data collection and analysis

Demographic and clinical characteristics of mothers and neonates as well as neonatal outcomes were extracted from charts into a pretested data collection form. Data were then transferred into an Access electronic database. Travel time from health center to district hospital was calculated using an average estimate of the time for an ambulance to travel from the mother’s health center of first presentation to the district hospital. We could not obtain an actual measure for a specific mother’s travel time as that information is not routinely collected. The severity of the indication for cesarean section was classified into two categories, very severe and severe, based on complication to the fetus and through consultation with a local obstetrician. Very severe indication included uterine rupture, fetal distress, cord prolapse, and abruption placenta and severe indication included preeclampsia, prolonged rupture of membranes, cephalo-pelvic disproportion, prolonged labor, and mal-presentation. In the case of more than one indication, we categorized according to the most severe indication. Neonatal outcome was dichotomized into poor neonatal outcome, defined as either death within 24 h or an APGAR score less than 7 at 5 min, or good neonatal outcome, defined as alive with an APGAR greater than or equal to 7 at 5 min.

We described mothers’ demographic and clinical characteristics using frequencies and percentages for categorical data. We tested each variable’s relationship to neonatal outcome using a Chi-squared test or in the case of small cell counts, a Fisher’s exact test. Factors significant at α = 0.20 level were retained for consideration in the multivariate logistic regression model. We developed the final model using backward stepwise selection, such that only factors significant at α = 0.05 significance level were retained in the final model. We report odds ratios (ORs), 95% confidence intervals (95%CIs) and p-values. For univariate and bivariate analyses, missing data were ignored, but we reported a reduced sample size in the case of missing data. For multivariate analysis, if a variable was missing for 15% or more observations, then we included a missing category in the model. All analyses considered the neonate as the unit of analysis and were completed in Stata v14.0 (College Station, TX: StataCorp LP).

## Results

**Fig. 1:**
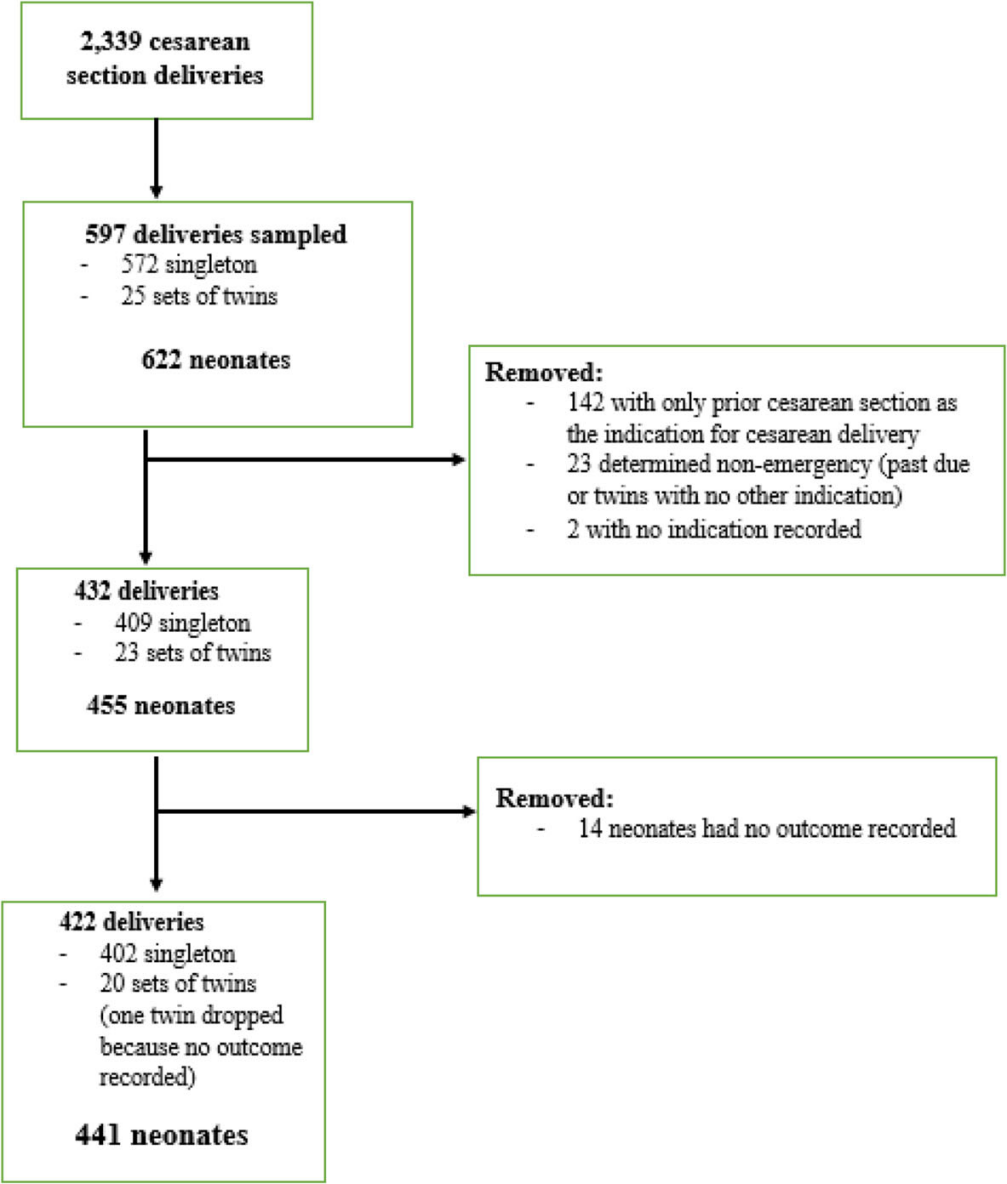
Flow diagram of chart review

In 2015, a total of 2,339 mothers underwent emergency cesarean section, of which 597 were randomly sampled, corresponding to 622 neonates (Fig. 1). Of these, 422 deliveries, corresponding to 441 neonates, were confirmed to have emergent conditions and had neonatal outcome recorded. For the 435 neonates with mother’s ages recorded, 189 (43.4%) were born to mothers between 25–34 years old (Table 1). One hundred and eighty-three neonates (41.8% of 438) were born to women with 1–3 prior pregnancies. Most neonates had mothers with community-based health insurance (377 out of 415, 90.9%). For the 356 neonates with information about travel time from the health center to the district hospital, 91 (25.5%) were from health centers attached to a district hospital, 200 (56.1%) were from health centers between 30–60 min travel time to the district hospital, and 65 (18.2%) were from health centers with over 60 min travel time to get to the district hospital.

**Table 1:** Demographic characteristics of mothers undergoing emergency cesarean section delivery (*N* = 441)

Variable	Number	Percent
District hospital		
Butaro	161	36.5
Kirehe	139	31.5
Rwinkwavu	141	32.0
Mother’s age group	*N* = 435	
15–24	176	40.5
25–34	189	43.4
35+	70	16.1
Number of previous pregnancies	*N* = 438	
0	176	40.2
1–3	183	41.8
4+	79	18.0
Mother’s marital status	*N* = 404	
Single	32	7.9
Ever married	372	92.1
Mother’s weight	*N* = 365	
≤ 50 kg	24	6.6
51–80 kg	329	90.1
> 80 kg	12	3.3
Occupation	*N* = 409	
Farmer	373	91.2
Employed	22	5.4
Unemployed	14	3.4
Insurance	*N* = 415	
Community based health insurance	377	90.8
Private insurance	26	6.3
None	12	2.9
Travel time from health center to the hospital^a^	*N* = 356	
Health center attached to the hospital	91	25.6
30–60 min	200	56.2
> 60 min	65	18.2

^a^Travel time measured as average number of minutes for ambulance to travel from the health center to the district hospital

Of the 441 neonates, 402 (91.1%) were singleton births (Table 2). For the 336 neonates with gestational age recorded, 59 (16.1%) were less than 38 weeks of gestational age. One hundred and twenty-nine neonates (37.0% of 349) were born to mothers with a history of prior cesarean delivery. The most common indications for cesarean section included fetal distress (32.0%, 141) and prolonged labor (30.6%, 135) and for 203 (46.0%) neonates, their mothers had multiple indications for cesarean delivery. For 180 (40.8%) neonates, the mother presented with a very severe indication for emergency cesarean section and 40 (9.0%) neonates either died or had an APGAR < 7 at 5 min post-delivery.

**Table 2:** Clinical characteristics of mothers and neonatal outcomes (*N* = 441)

Clinical characteristics	Number	Percent
Number of fetuses		
1	402	91.2
2	39	8.8
Gestational age	*N* = 366	
< 38 weeks	59	16.1
38–42 weeks	284	77.6
> 42 weeks	23	6.3
Duration of contractions prior to admission	*N* = 279	
≤ 12 h	83	29.7
13–24 h	113	40.5
> 24 h	83	29.8
Fetal heart rate at admission (bpm)	*N* = 429	
< 120	59	13.8
120–160	352	82.1
> 160	18	4.2
Maternal blood pressure at admission (SBP/DBP)	*N* = 397	
Hypotensive (<90/<50)	2	0.5
Normal (90–140/50–90)	368	92.7
Hypertensive (>140/>90)	27	6.8
Oxygen saturation on admission	*N* = 216	
Low saturation (≤94%)	7	3.2
Normal saturation (>94%)	209	96.8
Hemoglobin level (grams/deciliter)	*N* = 285	
Low (<12)	48	16.8
Normal (12–16)	231	81.1
Above range (>16)	6	2.1
History of prior cesarean section	*N* = 349	
No	220	63.0
Yes	129	37.0
Indications for cesarean section		
Fetal distress	141	32.0
Prolonged labor	135	30.6
Prior cesarean section	129	29.3
Malpresentation	108	24.5
Cephalopelvic disproportion	69	15.6
Prolonged rupture of membranes	46	7.9
Placenta previa	21	4.8
Intra-uterine rupture	7	1.6
Cord prolapse	6	1.4
Preeclampsia	6	1.4
Abruption placenta	5	1.1
Number of indications per woman		
Single indications	238	54.0
2 indications	179	40.6
3+ indications	24	5.4
Severity of cesarean indication		
Very severe indication^a^	180	40.8
Severe indication^b^	261	59.2
Neonatal clinical outcome		
Alive and APGAR ≥ 7	401	91.0
Died, or APGAR < 7	40	9.0

*SBP* Systolic blood pressure

*DBP* Diastolic blood pressure

^a^Very severe indication was defined as intrauterine rupture, fetal distress, cord prolapse, and abruptio placentae

^b^Severe indication was defined as preeclampsia, prolonged rupture of membranes, cephalo-pelvic disproportion, prolonged labor and mal-presentation

In the bivariate analysis, five maternal factors were associated with neonatal outcomes (Table 3). These included number of prior pregnancies (*p* = 0.057), mother’s weight (*p* = 0.060), travel time from health center to district hospital (*p* = 0.022), number of fetuses (*p* = 0.148), and severity of cesarean section indication (*p* = 0.064). For the multivariate analysis, three of these factors remained significantly associated with neonatal outcomes (Table 4). Neonates born to mothers with four or more prior deliveries had higher odds of having a poor outcome as compared to neonates of mothers with 1–3 prior deliveries (OR = 3.01, 95% CI: 1.23, 7.35, *p* = 0.015). A longer ambulance travel time from health center to hospital was associated with worse neonatal outcomes compared to neonates of mothers coming from health centers attached to the district hospital (OR = 3.80, 95% CI: 1.07, 13.40, *p* = 0.038 for 30–60 min and OR = 5.82, 95% CI: 1.47, 23.05, *p* = 0.012 for 60 min or more). Neonates whose mothers presented with very severe indication had significantly worse outcomes compared to those whose mothers had severe indication (OR = 2.24, 95% CI: 1.11, 4.52, *p* = 0.023).

**Table 3:** Bivariate analysis of neonatal outcomes

	Alive and APGAR ≥ 7 (*N* = 401)		Died, or APGAR < 7 (*N* = 40)		
	n	%	n	%	*p*-value
District hospital					
Butaro	147	91.3	14	8.7	0.486
Kirehe	128	92.2	10	7.2	
Rwinkwavu	125	88.7	16	11.3	
Mother’s age group	(*N* = 395)				
15–24	163	92.6	13	7.4	0.392
25–34	171	90.5	18	9.5	
35+	61	87.1	9	12.9	
Number of prior pregnancies			(*N* = 37)		
0	162	92.1	14	87.9	0.057
1–3	172	94.0	11	6.0	
4+	67	84.8	12	15.2	
History of prior cesarean section	(*N* = 320)		(*N* = 29)		
No	199	90.5	21	9.5	0.320
Yes	121	93.8	8	6.2	
Mother’s marital status	(*N* = 371)		(*N* = 33)		
Single	28	87.5	4	12.5	0.317
Ever married	343	92.2	29	7.8	
Mother’s weight	(*N* = 333)		(*N* = 32)		
≤ 50 kg	21	87.5	3	12.5	0.060
51–80 kg	303	92.1	31	7.9	
> 80 kg	9	75.0	3	25.0	
Occupation	(*N* = 372)		(*N* = 37)		
Farmer	338	90.6	35	9.3	0.261
Employed	22	100.0	0	0.0	
Unemployed	12	85.7	2	14.3	
Insurance	(*N* = 379)		(*N* = 36)		
Community based health insurance	344	91.2	33	8.8	>0.999
Private insurance	24	92.3	2	7.7	
None	11	91.7	1	8.3	
Travel time from health center to the hospital^c^	(*N* = 321)		(*N* = 37)		
Health center attached to the hospital	88	96.7	5	3.3	0.022
30–60 min	178	89.0	22	11.0	
> 60 min	55	84.6	10	15.4	
Number of fetuses			(*N* = 39)		
1	368	91.5	33	8.5	0.148
2	33	84.6	6	15.4	
Gestational age					
< 38 weeks	54	91.5	5	8.5	0.936
38–42 weeks	262	92.2	22	7.8	
> 42 weeks	85	86.7	13	13.3	
Duration of contractions prior to admission	(*N* = 258)		(*N* = 21)		
≤ 12 h	75	90.4	8	9.6	0.656
13–24 h	106	93.8	7	6.2	
> 24 h	77	92.8	6	7.2	
Maternal blood pressure at admission (SBP/DBP)	(*N* = 365)		(*N* = 32)		
Hypotension (<90/<50)	2	100.0	0	0.0	0.374
Normal (90–140/50–90)	340	92.4	28	7.6	
Hypertension (>140/>90)	23	85.2	4	14.8	
Oxygen saturation on admission	(*N* = 195)		(*N* = 21)		
Low saturation (≤94%)	6	85.7	1	14.3	0.516
Normal saturation (>94%)	189	90.4	20	9.6	
Hemoglobin level (grams/deciliter)	(*N* = 254)		(*N* = 31)		
Low (<12)	42	87.5	6	12.5	0.582
Normal (12–16)	207	89.6	24	10.4	
Above range (>16)	5	83.3	1	16.7	
Severity of cesarean section indication					
Very severe indication^a^	158	87.8	22	12.2	0.064
Severe indication^b^	243	93.1	18	6.9	

*SBP* Systolic blood pressure

*DBP* Diastolic blood pressure

^a^Very severe indication included intrauterine rupture, fetal distress, cord prolapse and abruptio placentae

^b^Severe indication was defined as preeclampsia, prolonged rupture of membranes, cephalo-pelvic disproportion, prolonged labor and mal-presentation

^c^Travel time measured as average number of minutes for ambulance to travel from the health center to the district hospital

**Table 4:** Multivariate analysis of maternal factors as predictors of neonatal outcomes

Variables	OR	95% CI	*p*-value
Number of pregnancies			
0	1.40	[0.60, 3.23]	0.431
1–3	Ref	-	-
4+	3.01	[1.23, 7.35]	0.015
Travel time from the health center to the hospital^c^			
Health center attached to the hospital	Ref	-	-
30–60 min	3.80	[1.07, 13.40]	0.038
> 60 min	5.82	[1.47, 23.05]	0.012
Unknown	2.12	[0.48, 9.35]	0.317
Severity of cesarean section indication			
Very severe indication^a^	2.24	[1.11, 4.52]	0.023
Severe indication^b^	Ref	-	-

^a^Very severe indication included intrauterine rupture, fetal distress, cord prolapse, abruptio placentae

^b^Severe indication was defined as preeclampsia, prolonged rupture of membranes, cephalo-pelvic disproportion, prolonged labor and mal-presentation

^c^Travel time measured as average number of minutes for ambulance to travel from the health center to the district hospital

## Discussion

Our study included 441 neonates born via emergency cesarean delivery, of which 9.0% died or had an APGAR score of less than seven at 5 min. This is comparable to outcomes in Africa where a study by the World Health Organization showed an average neonatal death rate of 12.9% after cesarean section [3]. Poor neonatal outcomes were associated with a mother having four or more previous deliveries, longer travel times from the health center to the district hospital, and having a very severe indication for cesarean delivery. The latter result is not surprising, given that the creation of this category was based on previous knowledge of associated risks for certain complications [15]. However, we recommend continued sensitization and support for health center nurses, for example through the emergency obstetric conditions training [8] or the maternal health mentorship program [16] as well as improving health center diagnostic capacities to quickly identify very severe indications so as to provide timely referral.

Previous studies in sub-Saharan Africa have linked longer travel distance to a main road or to the hospital with higher risks of mortality for neonates and children under five years [17, 18]. Rwandan Ministry of Health’s 2014 Health Sector Policy emphasized the decentralization of health facilities with a target to ensure that all people have access to a health center within 5 km of their homes [19]. However, currently health centers lack staff and facilities and are not mandated by Rwanda Ministry of Health to perform cesarean sections and there are only one or two district hospitals in each district, which leads to women having to travel longer distances to seek emergency obstetric care. Moreover, poor and difficult road networks were shown to be associated with delay in reaching health facility where needed level of care is available and contribute to poor neonatal outcome [20]. Thus, the variable considered in this study, travel time from the health center to the district hospital, while not the exact measure of the woman’s travel to time to the hospital and likely underestimating the total travel time, is of particular importance in this context, since a pregnant woman must go first to her catchment area health center prior to going to the district hospital. Ambulances should be used to transport emergency patients from health centers to the district hospital, but the availability of ambulances is not always confirmed. Improving road networks would lessen difficulties and travel time to district hospitals for laboring mothers. In the short term, we recommend an improved referral system for emergency obstetric cases as well as ensuring the availability of ambulances at remote health centers to shorten travel times and improve neonatal outcome. In the long run, increasing the number of hospitals per district with facilities and staff to provide emergency obstetric care may reduce travel time and hence the negative effect of longer travels time on neonatal outcome.

Our finding that neonates born to mothers with four or more previous pregnancies were more likely to have bad outcomes is consistent with a study conducted in Nigeria [21]. Although this could reflect the association between increased number of pregnancies and advanced maternal age, which is an established risk factor for poor neonatal outcomes in general [22–24], our analyses that explored mother’s age directly did not find an association between maternal age and neonatal outcome. Women who have many pregnancies are often times poorer and less educated [25], and both poverty and low education have been linked to poor neonatal outcomes in sub-Saharan Africa [26]. Finally, previous pregnancies can lead to faster progression of labor [27], and this may lead to precipitated labor that can negatively affect neonatal outcome, if the cesarean section is not immediately available. The link between previous number of deliveries and risk for poor neonatal outcomes among emergency cesarean deliveries should be studied further.

There are several limitations to be considered in this study. Given that this study was retrospective and relied on routinely collected clinical data, some variables such as height of the mother to determine body mass index, income, education levels, nutrition status and tobacco or alcohol use were not available for analysis. Additionally, some of our existing variables had missing data from the chart review. Unfortunately, inadequate documentation is often seen in these settings, and proper documentation should be encouraged for clinical care and future studies. Finally, this study was conducted at district hospitals supported by Partners In Health, whereas other hospitals do not receive this support. However, the population profile and pathways to cesarean delivery are similar to other district hospitals in Rwanda and sub-Saharan Africa and therefore we believe the results are generalizable beyond this population.

## Conclusions

Poor neonatal outcomes were linked to longer travel time to district hospitals, mothers having multiple previous pregnancies, and a very severe indication for cesarean delivery. While it is infeasible to provide cesarean sections in health centers in the near future, increasing the number of district hospitals and improving timely access to transport to district hospitals should be considered. Furthermore, it is important to increase the capacity of health centers to diagnose very severe indications for cesarean delivery, and provide regular mentorship to the health center nurses so as to improve time to intervention. Future studies should assess effect of fetal factors and time delays and compare outcomes between cesarean and vaginal deliveries.

## Abbreviations

APGAR: Appearance, pulse, grimace, activity, respiration
CI: Confidence interval
IRB: Institutional review board
MOH: Ministry of health
OR: Odds ratio
P: P value
RDHS: Rwanda demographic health survey.
